# Effects of Reading Proficiency and of Base and Whole-Word Frequency on Reading Noun- and Verb-Derived Words: An Eye-Tracking Study in Italian Primary School Children

**DOI:** 10.3389/fpsyg.2018.02335

**Published:** 2018-11-28

**Authors:** Daniela Traficante, Marco Marelli, Claudio Luzzatti

**Affiliations:** ^1^Department of Psychology, Catholic University of Milan, Milan, Italy; ^2^NeuroMI – Milan Centre for Neuroscience, Milan, Italy; ^3^Department of Psychology, University of Milano-Bicocca, Milan, Italy

**Keywords:** reading acquisition, word morphology, eye movements, verb-derived nouns, noun-derived nouns, word frequency, reading proficiency, derived words

## Abstract

The aim of this study is to assess the role of readers’ proficiency and of the base-word distributional properties on eye-movement behavior. Sixty-two typically developing children, attending 3^rd^, 4^th^, and 5^th^ grade, were asked to read derived words in a sentence context. Target words were nouns derived from noun bases (e.g., *umorista*, ‘humorist’), which in Italian are shared by few derived words, and nouns derived from verb bases (e.g., *punizione*, ‘punishment’), which are shared by about 50 different inflected forms and several derived words. Data shows that base and word frequency affected first-fixation duration for nouns derived from noun bases, but in an opposite way: base frequency had a facilitative effect on first fixation, whereas word frequency exerted an inhibitory effect. These results were interpreted as a competition between early accessed base words (e.g., *camino*, chimney) and target words (e.g., *caminetto*, fireplace). For nouns derived from verb bases, an inhibitory base frequency effect but no word frequency effect was observed. These results suggest that syntactic context, calling for a noun in the target position, lead to an inhibitory effect when a verb base was detected, and made it difficult for readers to access the corresponding base+suffix combination (whole word) in the very early processing phases. Gaze duration was mainly affected by word frequency and length: for nouns derived from noun bases, this interaction was modulated by proficiency, as length effect was stronger for less proficient readers, while they were processing low-frequency words. For nouns derived from verb bases, though, all children, irrespective of their reading ability, showed sensitivity to the interaction within frequency of base+suffix combination (word frequency) and target length. Results of this study are consistent with those of other Italian studies that contrasted noun and verb processing, and confirm that distributional properties of morphemic constituents have a significant impact on the strategies used for processing morphologically complex words.

## Introduction

Over the past 40 years (see Clifton et al., 2016 for a review), several studies on eye movements found that information uptake is graded, involving the extraction of graphemic details near fixation, and of coarse-grained information from the parafovea. Furthermore, Rayner’s work (e.g., Liversedge et al., 2004; Dambacher et al., 2013) demonstrated that “experienced readers look essentially at every word in text, and that the ability to extract visual information quickly and efficiently is the foundation of skilled reading” (Clifton et al., 2016, p. 2). Research carried out in this field provides strong evidence that linguistic processing primarily influences eye movements during reading. In particular, Rayner and co-workers demonstrated that three major lexical and semantic properties influence how easily written words are processed: word frequency (e.g., Rayner and Duffy, 1986), word length (e.g., Juhasz et al., 2008), and predictability in context (e.g., Ehrlich and Rayner, 1981) (“the big three” of lexical processing).

The deep relationship between lexical processing and eye-movement behavior, proved by the studies that investigated eye-movements in reading, had great consequence on research strategy: the more the way lexical processing influences the eye-movement behavior is learned, the more it is possible to use eye-movement measures to investigate basic language processing. In this research field, several variables are analyzed, but the main variables, considered measures of initial and spatially localized processing, are first-fixation duration and gaze duration (Liversedge et al., 1998). The former is the time the reader initially spends fixating the target word, and “it is generally taken to be the very earliest point at which we might expect to see an effect due to experimental manipulation” (Liversedge et al., 1998, p. 58). Gaze duration “is defined as the sum of all the fixations made in a region until the point of fixation leaves the region” (*ibidem*). “Visual (e.g., the amount of contrast, font size), lexical (e.g., frequency), and contextual (e.g., predictability) properties of words yield a graded effect on fixation durations (e.g., as word frequency decreases, fixation durations increase)” (Dambacher et al., 2013, p. 1468).

For what concerns morphologically complex words, “eye movements can give a clear insight into the time course of morphological processing” (Bertram, 2011, p. 92). In particular, the first of multiple fixations on the target word is likely to reflect an early processing stage, whereas gaze duration can be considered an index of later processing (Bertram, 2011). Studies on eye movements in reading Finnish and English compounds embedded in sentences (Hyönä and Pollatsek, 1998; Pollatsek et al., 2000, 2011; Andrews et al., 2004; Pollatsek and Hyönä, 2005) found a significant effect of first-constituent frequency on first-fixation duration, whereas gaze duration was affected by the frequency of both first and second constituents, as well as by whole word frequency. The general assumption of studies involving morphologically complex words is that the first-constituent frequency effect is evidence of a decomposition process; consequently, first-fixation duration is likely to be involved in early morphemic parsing. As for whole-word effects, they are usually considered to be diagnostic of whole-word access. So, it can be assumed that gaze duration taps into lexical access. Concurrent whole-word and morphological effects are evidence of the fact that storage and computation interact in complex word recognition.

Data from more recent research shows a more complex pattern of results on the variables affecting early stages of compound processing. Bertram and Hyönä (2003) found an effect of compound-word length and proposed that when long compounds are processed, first-fixation duration is affected by first constituent frequency. On the contrary, when reading short compounds, readers start to analyze the whole-word string, so the whole-word frequency effect emerges. Studies on Dutch compounds (Kuperman et al., 2008, 2009) pointed out the role of the interaction between compound frequency and first-constituent frequency, as they showed that the higher the whole-word frequency of a compound the smaller the role of the first-constituent frequency in first-fixation duration. Marelli and Luzzatti (2012) data confirmed this interaction in Italian, and showed that the first-constituent frequency effect is also modulated by the semantic transparency of the compound: the higher the semantic transparency is, the greater the facilitation effect of first-constituent frequency on first-fixation duration. More recently, the role of semantic transparency in processing derived words was pointed out by Amenta et al. (2015): their results showed that stem frequency has a facilitating effect on first-fixation duration only when the target word is embedded in sentences prompting a semantically transparent interpretation of the word, whereas a stem-frequency effect is inhibitory when the sentence context prompts an opaque interpretation of the target word.

Data supporting this complex pattern of results also come from studies on derived words. Niswander-Klement and Pollatsek (2006) found root frequency effects only for long prefixed words (about eight letters long), but not for short prefixed words (about six letters long). In contrast, whole-word frequency effects were found for short prefixed words, but not for the long ones. Consistently with the previous results, Kuperman et al. (2010) found an interaction between whole-word frequency and length of the suffix in a large regression study on Dutch derived words: the shorter the suffix is, the stronger the whole-word frequency effect on reading times. Furthermore, the study found that also the amount of information carried by the word-base and the size of the morphological family of the suffix affect the reading times. The authors interpreted the latter result as a *relative entropy* effect of the morphemes embedded in the morphologically complex words. In particular, words containing most salient suffixes, i.e., affixes that occur in a larger and more frequent number of words, are processed faster.

Evidence from eye-movement research mirrors what has been reported in several studies, employing different experimental procedures, in languages with rich morphology systems. Kostić and Katz (1987), in Serbo-Croatian, found differences in lexical decision times for nouns, adjectives, and verbs: the authors referred this effect to the number of inflectional alternatives available for each grammatical class. Differences in processing verbs and nouns were found also in Hebrew (Deutsch et al., 1998). This evidence led the authors to suggest that, beyond semantic and syntactic components, the distributional properties of constituents may play a significant role in word processing: “When a morpheme is common to more words in the language, its impact on processes of morphological decomposition is prominent” (Deutsch et al., 1998, p. 1252).

The Italian language can be very informative in testing this claim, as verb roots are shared by about 50 different inflected forms and several derived words (e.g., *punizione*, ‘punishment’ from *punire*, ‘to punish’), whereas noun roots can be inflected in 2 to 4 forms and are shared by fewer derived words. For this reason, it may be expected that verb processing is a more demanding task than noun processing, as the identification of the target word within a large morphological family is likely to require more difficult selection processes than identification within a small morphological family.

In Italian, skilled adult readers tend to recognize and read aloud verbs more slowly than nouns and adjectives. Moreover, latency for verbs, but not for nouns or adjectives, is correlated with their base frequency (Colombo and Burani, 2002; Traficante and Burani, 2003). Differences in processing nouns and verbs were also found in several studies on adults suffering from acquired language disorders (Zingeser and Berndt, 1990; Chen and Bates, 1998; Luzzatti et al., 2002; Crepaldi et al., 2006; see for a review Crepaldi et al., 2011). However, some studies showed that verb and noun processing were deeply influenced also by type of task and experimental paradigm: even noun production can be more difficult than verb production in a Grammatical-Class Switching Task^1^ (GCST), both in skilled adult readers (Marangolo et al., 2006; Berlingeri et al., 2008) and in people suffering from Parkinson’s Disease (Di Tella et al., 2018; Silveri et al., 2018). Data from fMRI, reported in these studies, shows that the grammatical category (either verbs or nouns), which is associated to the longest reaction times, triggers a greater activation of the left inferior frontal gyrus. In other words, it seems that processing difficulties cannot be referred to grammatical category as such, but to the complexity of selection and inhibition processes required by the task.

The complex system for extracting information from written words develops during reading acquisition. So, in the early phases of learning to read, children show a pattern of eye-movements that is only partially similar to the pattern observed in skilled adult readers: they tend to make longer fixations and shorter saccades, with more frequent regressions to earlier parts of the text, and are more likely to re-fixate in proximity to the end of the word (see Bellocchi et al., 2013 for a review). Moreover, several studies showed that children have a smaller perceptual span, i.e., Moreover, several previous studies in the “region from which useful information can be obtained during fixation in reading” (Rayner, 1986, p. 212). Due to their reduced perceptual span, children are supposed to be prone to process morphologically complex stimuli through morphemic constituents, even in the case of short complex words, in order to acquire as much information as possible during a single fixation (Bertram and Hyönä, 2003). Häikiö et al. (2011) studied the role of morphology in reading acquisition by measuring children’s eye movements while reading sentences containing either a hyphenated (e.g., *ulko-ovi*, ‘front door’) or concatenated (e.g., *autopeli*, ‘racing game’) compound. The participants were Finnish second, fourth, and sixth graders (8, 10, and 12 years old, respectively). Fast second-graders and all fourth- and sixth-graders read concatenated compounds faster than hyphenated compounds: this suggests that they prefer to process concatenated compounds via whole-word representations. In contrast, fixation durations of slow-reading children attending second grade were shorter for hyphenated than concatenated compounds. Such a result supports the idea that these children process all compounds via constituent morphemes and that hyphenation comes to aid in the process. Häikiö et al. (2011) results show that the lower the reading ability, the higher the probability of morphemic parsing.

Grainger and Ziegler (2011) model offers a useful framework to describe the role of morphemic representations during reading acquisition. According to this model, readers who are beginners start from learning associations between letters and sounds: orthographic input is processed letter-by-letter, in a laborious serial procedure of phonological recoding. Then, the repeated exposure to printed words leads to the development of two different types of orthographic codes, fine- and coarse-grained representations, that optimize the mapping from letters to meaning. Letters that co-occur very often in a language, which is typically the case for morphemic constituents (e.g., ‘-er’), can be grouped in chunks, forming higher-level orthographic representations, in which the information about the ordering of letters in the string is preserved (fine-grained orthography). Chunking leads to an improvement in reading performance, as it reduces the number of units to be processed. However, to speed up the process further, skilled readers learn to process the string of letters only for uptaking information about the presence of letter combinations without precise positional information (coarse-grained orthography). In this way the mapping between printed word and meaning is realized ‘at a glance,’ according to distributional properties of the word features in the language (diagnosticity).

Indeed, data from developmental literature proved that the presence of morphemic constituents may help word processing in struggling readers and in skilled young readers when either low-frequency words or pseudowords must be read. This suggests that morphemes may act as distributional cues that can be efficiently exploited to facilitate reading (Mann and Singson, 2003; Carlisle and Stone, 2005; Burani et al., 2008; Jarmulowicz et al., 2008; Burani, 2010; Deacon et al., 2011; Marcolini et al., 2011; Traficante et al., 2011; Traficante, 2012; Deacon and Francis, 2017). However, helpfulness of morphemic constituents may vary according to the consistency of grapheme-to-phoneme correspondence and of morphological richness of the language.

Casalis et al. (2015), through a direct comparison between English and French, found that, in a lexical decision task, the recognition of a root [R] within a word tends to slow down the response time, irrespective of the presence of a suffix [S] (e.g., *farmer* [R+S+], *window* [R+S-] > *murder* [R-S+], *narrow* [R-S-]) only in English 4^th^ grade children, whereas the same condition (e.g., *fermier*, *boutique*) did not lead to the same effect in French children, matched by grade with their English peers. On the contrary, in both languages, children are slower and less accurate in rejecting pseudowords containing a stem and/or a suffix. In Italian, a shallow orthography language with rich morphology, the facilitative role of root frequency in word reading has been found, in typically developing children, only for low-frequency words, whereas in poor readers it has been observed for both high- and low-frequency words (Marcolini et al., 2011). However, when reading aloud pseudowords, all Italian children, irrespective of their reading skills, gained advantage in latency for the presence of a root (e.g., ^∗^*bagnezza* [R+S+], ^∗^*bagnezzo* [R+S-] < ^∗^*bognezza* [R-S+], ^∗^*bognezzo* [R-S-]) and increased their accuracy from the presence of both a root and a suffix (i.e., ^∗^*bagnezza*, ^∗^*bagnezzo*, ^∗^*bognezza* > ^∗^*bognezzo*) (Traficante et al., 2011).

Recently, we assessed the role of distributional properties of morphemic constituents on reading words, by asking 4^th^ and 5^th^ graders to read aloud nouns derived from verb- and from noun-bases. Latency data showed that roots occurring in many word forms, like verb roots, are more likely to trigger morphemic parsing than roots embedded in few forms, like noun roots (Traficante et al., 2014; see also Traficante and Burani, 2003). In fact, in a reading-aloud task, base frequency and word frequency effects were found only for words derived from verb bases (e.g., ‘*fallimento*,’ failure). The base frequency effect seemed to suggest that young readers (4^th^ and 5^th^ graders) exploit verb bases as a head-start for word naming; however, in a task involving input and output processes, the advantage of such a head-start might be counterbalanced by the low probability of finding the specific ‘base+suffix’ combination. As a consequence, for deverbal items a strong word frequency effect emerged: the higher the frequency of the ‘base+suffix’ combination (i.e., word frequency, see Baayen, 2010), the faster the onset of the response. However, words derived from verb bases were read more slowly than words derived from noun bases, thus confirming that the parsing procedure is time-costly. For nouns embedding noun bases (e.g., ‘*artista*,’ artist) no lexical effect, but only word length effect was detected on RTs. This result was interpreted as the effect of a sublexical strategy applied by Italian children in reading low-frequency words derived from a noun base, which would not trigger morphemic parsing. Data on accuracy confirmed that reading nouns derived from verb bases is more difficult than reading nouns derived from noun bases, but showed a different picture on the role of morpho-lexical variables. In fact, both base frequency and whole-word frequency influenced reading accuracy, irrespective of the base-word category: higher frequency values were associated with higher accuracy not only for nouns derived from a verb, but also for nouns derived from a noun. These results on accuracy confirm that verb bases trigger morphemic parsing, but suggest that also noun bases and whole-word representations of words derived from noun bases should be accessed at some point of the word processing.

To better analyze the processes involved in reading derived words, in the present study we analyzed eye-movements in reading aloud the same derived words used in Traficante et al. (2014) study. In this case they were embedded in sentences, according to the experimental procedure usually adopted in eye-movements studies. Considering the above-mentioned literature on eye movements in reading complex words, this technique is expected to allow us to directly assess the role of base word as a head-start in early processing stages (through the testing of base-word frequency effect on first-fixation duration) and to get some clue on the later processing stages (through the analysis of base- and whole-word effects on gaze-duration, which should mirror reading latency). As for nouns derived from verb bases, it was expected to find, from eye-movement analysis, data confirming the use of morphemic constituents in word recognition, i.e., morpho-lexical effects on both first-fixation duration and gaze duration. Yet, for nouns derived from noun bases, contrasting results from latency and accuracy observed in the previous study make predictions less clear. On one hand, the presence of morpho-lexical effects on accuracy suggests that representations of morphemic constituents should play a role in programming pronunciation of the target word; as a consequence, base and whole-word frequency are expected to influence at least gaze duration. On the other hand, the lack of morpho-lexical effects on latency, which was affected only by length in letters, might be due to complex effects, on which eye-movements analysis is supposed to shed light.

Finally, we chose to consider the role of proficiency in reading, beyond the school grade attended by children, because Häikiö et al. (2011) results suggest that, in early stages of learning to read, orthographic skills can modulate the use of morphemic constituents, irrespective of grade. In this vein, also Beyersmann et al. (2015) showed that morphological effects were modulated by reading proficiency, in a masked-priming paradigm, and not by grade. Moreover, several previous studies in Italian (e.g., Marcolini et al., 2011) found that poor readers are more prone to use morphemic parsing than skilled readers, even though attending the same school grade. The prediction for the present study is that the lower the proficiency, the more likely it is to observe lexical effects on first fixation duration, as a consequence of using a base word as a head start in early processing of the target word, before accessing the whole word.

## Materials and Methods

### Participants

Sixty-two children (35 Female; 56%), attending 3^rd^ (*N* = 20), 4^th^ (*N* = 22), and 5^th^ grade (*N* = 20) (mean age = 8.98 years; *SD* = 0.81) were recruited in three different primary schools in Northern Italy. All children were born in Italy, attended school regularly and had normal or corrected-to-normal vision. None of them had been signaled for neurodevelopmental disorders according to DSM-5 (American Psychiatric Association, 2013).

The present study was approved by the Scientific and Ethics Committee of the Department of Psychology of Catholic University of Milan, in accordance with the Helsinki Declaration and all children’s parents gave informed written consent to the study.

### Materials

#### Experimental Stimuli

Seventy suffixed nouns (see Traficante et al., 2014) were selected from the derived words listed in the *Elementary lexicon: Statistical data on written and read Italian language in primary school children* (Marconi et al., 1993), according to the following criteria: (i) having the base word listed in the *Elementary lexicon*; (ii) bearing a semantically transparent derivation, according to a rating scale administered to 20 undergraduate students (*M* = 4.9, range = 2.4–6.6 on a 1–7 Likert scale); (iii) being phonologically transparent with respect to its base; (iv) ending with frequent and productive derivational suffixes; (v) being stressed on the penultimate syllable. Only words derived from noun-base or verb-base were selected. As a result, two sets of derived words were obtained: 41 nouns derived from noun-base (e.g., ‘artista’, artist), and 29 nouns derived from verb-base (e.g., ‘punizione’, punishment) (Table 1).

**Table 1:** Mean and standard deviations (in parentheses) for the main psycholinguistic features of the stimuli.

		Nouns derived from noun bases (*N* = 41) (e.g., *artista*, artist)	Nouns derived from verb bases (*N* = 29) (e.g., *creazione*, creation)
Number of forms in the inflectional paradigm of the base		2–4	>50
Word length	*M*	8.07	9.48
	*SD*	(1.1)	(1.5)
Word frequency^a^	*M*	13.78	37.75
	*SD*	(16.0)	(39.1)
Root length	*M*	4.41	4.97
	*SD*	(1.1)	(1.3)
Base frequency ^a^	*M*	102.21	141.28
	*SD*	(107.76)	(124.5)

*^a^Frequency measures are calculated on 1 million occurrences from a corpus of written Italian (Bertinetto et al., 2005).*

A brief sentence (mean length in characters = 47.04; minimum = 32, maximum = 53) was generated for each target word, following two main criteria: (i) the position of the target word had to be in the middle of the sentence; (ii) the predictability of the target word in the sentence context was low (see Supplementary Material).

In order to exclude a difference in the extent to which target words were predictable given the preceding context, we estimated their contextual plausibility by means of distributional systems. These types of computational models, widely used in cognitive science (a famous example being *Latent Semantic Analysis*; Landauer and Dumais, 1997), characterize words as semantic vectors, in turn induced by an analysis of lexical co-occurrences in text corpora. For the present purposes, we applied the WEISS2 system developed for Italian by Marelli (2017) using the word2vec learning algorithm (Mikolov et al., 2013). The representations for sentence contexts were obtained by summing the vectors of the words they include, following an established tradition in computational semantics (Mitchell and Lapata, 2010). Contextual plausibility was then computed as the cosine proximity between the context vector and the target-word vector: the higher this value, the more plausible the target word will be, given the preceding context. These estimates resulted to be similar in noun-base targets (*M* = 0.175, *SD* = 0.082) *vis-a-vis* verb-base targets (*M* = 0.140, *SD* = 0.081). Indeed, the contextual-plausibility distribution between the targets of either category was not significantly different (*p* = 0.18 at the Kolmogorv–Smirnoff test).

#### Word and Non-word Reading

To assess children’s proficiency in reading, the *Word and Non-word Reading Test* (Zoccolotti et al., 2005) was administered. It is a standard test made up of two lists of non-words (short: 4–5 letters; long: 8–10 letters) and four lists of words, varied in length (short: 4–5 letters; long: 8–10 letters) and in frequency (high and low frequency). Speed and accuracy in reading are assessed according to national norms.

#### Non-verbal Intelligence

Raven’s *Colored Progressive Matrices* (Raven, 1947; Italian adaptation, Belacchi et al., 2008) were administered to assess non-verbal reasoning abilities. All participants showed a performance within normative range.

### Apparatus

Each sentence was displayed on a 22-inch monitor, connected to a Dell Notebook PC W76CU interfaced with an SMI (Senso- Motoric Instruments) RED500 device, having high spatial (<0.4° of visual angle) and temporal (500 Hz) resolution. Viewing was binocular and the experiment was implemented and run through Experiment Center 3.0 software (2010).

### Procedure

Participants were seated approximately 60 cm from the monitor and the eye-tracker was calibrated by asking the children to track a black dot moving on the screen through nine different locations. Data on gaze accuracy measured during the calibration phase showed an average error on the horizontal axis of 0.43 degree between the actual gaze point and the point measured by the eye-tracker, approximately in line with the technical features declared by SMI for RED-500 device.

After setting the best calibration for each participant, they were asked to read aloud the sentences displayed in the middle of the screen. Sentences appeared centered in a single row, in black mono-spaced font (Consolas 24), in lower-case, on a white background and were presented one at a time in random order. Eighteen sentences were followed by a comprehension question with a multiple choice response. Reading was recorded and the rate of presentation was manually adapted by the experimenter according to the reading speed of each individual. Accuracy in reading target words was coded after transcription of the children’s productions.

### Data Analysis

To get an index of proficiency in reading, a *factor* analysis was performed on the reading speed measured on the six lists of the *Word and Non-word reading test* (Zoccolotti et al., 2005). A one-factor solution emerged, with a unique factor loading higher than 1 (λ = 4.78) and with 79.68% of explained variance. Factor scores were saved as a new variable, proficiency, negatively oriented: the higher the value, the lower the proficiency, as it derives from reading times. In all the analyses, the role of this variable was tested over and above the role of grade, in order to disentangle the effect of literacy exposure and explicit learning (grade) from the effect of reading skills and implicit learning (proficiency).

The raw eye-movement data was processed using Be-Gaze 3.0 software (2011). Only data from target words that were correctly read and that received at least 1 fixation was considered. For each target word two measures were analyzed: (i) First-fixation duration (FFD), i.e., the duration of the first of several fixations; (ii) Gaze duration (GD), i.e., the sum of all first-pass fixations. The first measure has been traditionally referred to early stages of processing, the latter to later stages of processing.

As we were interested in testing the effects of several psycholinguistic characteristics of the items, in interaction with individual differences within participants in reading skills, a linear multiple regression analysis approach was adopted. Mixed-effects models (Baayen et al., 2008), the most updated statistical procedure applied in the field, which offers the opportunity to take into account the random effects due to variability within participants and items, were used to analyze the fixed effects of proficiency (factor scores), base-word category (“noun” and “verb”), word length (standardized measure of length in letters), word frequency (logarithmically transformed), root length (standardized residuals from the regression of root length on word length), and base frequency (logarithmically transformed) on FFD and GD (both measures were logarithmically transformed). Analyses were carried out by means of R (R Development Core Team, 2009), and implemented in the lme4 R package (Bates et al., 2015). Subjects and items were considered as random intercepts. All mixed-effects models were tested with a model-criticism procedure, excluding outlier points on the basis of standardized residuals (with 2.5 *SD* as threshold) (Baayen, 2008). Statistics of the refitted models are reported. The *p*-values were computed adopting the Satterthwaite approximation for degrees of freedom (Satterthwaite, 1946) as implemented in the lmerTest R package (Kuznetsova et al., 2015).

Effects of psycholinguistic features on accuracy, computed as the proportion of correct responses after removing technical failures, were analyzed through Generalized linear mixed model fit by maximum likelihood (Laplace Approximation) (Jaeger, 2008).

*Post hoc* probing of significant interactions between categorical (noun vs. verb base) and continuous variables (word length, word frequency, root length, and base frequency) was carried out, according to Aiken and West (1991) suggestion: “because one of the predictor variables is categorical, the simple slopes of interest will be those evaluated at values of the dummy (or effect) variables that correspond to the separate groups” (Aiken and West, 1991, p. 130).

## Results

Only 2.6% of data points in which the eye-tracker did not record any fixation on the corresponding target word, were not considered in the analyses (see Brysbaert and Stevens, 2018 for power analysis in mixed-effects models).

### Accuracy

The total number of valid observations on which analyses on accuracy were carried out was 4250: 2486 for nouns derived from a noun-base and 1764 for nouns derived from a verb-base, respectively.

The rate of accuracy in reading target words was high (92%), thus confirming that in Italian typically developing children learn to read correctly in early school years, thanks to the high consistency of the grapheme-to-phoneme correspondence rules of the Italian language. However, it is worth noting that 5^th^ graders showed an accuracy rate (96%) higher than 3^rd^ (Tukey’s HSD: *p* = 0.048) and 4^th^ graders (Tukey’s HSD: *p* = 0.039) (90% of accuracy each) (*F*_2,59_ = 4.02, *p* = 0.023).

The analysis carried out by means of generalized linear mixed models, assessing the effects of grade and of all lexical variables (Model 1), showed the effect of grade (*b* = 0.5601, *z* = 2.630, *p* = 0.008) and word frequency (*b* = 0.1532, *z* = 1.97, *p* = 0.048) on accuracy: the higher the grade and word frequency the more accurate children’s production. However, an alternative model (Model 2), in which proficiency was added as covariate to the previous one^2^, showed a better fit index^3^ (AIC_model1_ = 2108.8, AIC_model2_ = 2099.8; *χ*^2^ = 10.978, *df* = 1, *p* < 0.001). In Model 2, grade effect did not reach significance level anymore (*b* = 0.1930, *z* = 0.892, *p* = 0.372), but proficiency was the strongest predictor of accuracy (*b* = -0.5865, *z* = -3.432, *p* < 0.001), and the role of word frequency was confirmed (*b* = 0.1559, *z* = 2.018, *p* = 0.043): the higher the proficiency^4^ and word frequency, the higher the accuracy.

### First-Fixation Duration

For the study of effects on FFD, only target words correctly read and undergoing more than one fixation were considered (*N* = 3627), as only in these cases FFD can be unambiguously referred to an early processing stage (Marelli and Luzzatti, 2012).

One-way ANOVA and *post hoc* analysis carried out on first fixation duration by grade (Table 2) showed a significant difference between 3^rd^ and 5^th^ graders (Tukey’s HSD: *p* = 0.041), whereas 4^th^ graders did not differ from the other children. Mean first-fixation duration was quite similar for the two types of derived words: for nouns derived from a noun base the mean duration was 218.3 ms (*SD* = 159.4), and for nouns derived from a verb base the mean duration was 225.3 ms (*SD* = 164.9).

**Table 2:** First fixation duration (ms): descriptive statistics and comparison within grades.

*Grade*	*N*	*M*	*SD*	*Minimum*	*Maximum*	*F*	*p*
3^rd^	20	246	77.3	145	386	3.13	0.051
4^th^	22	227	53.5	117	343		
5^th^	20	199	45.0	129	276		

A completely different picture turned out from mixed-effects models, in which grade and base-word category were considered in interaction with word length, word frequency, root length, and base frequency (Model 1). Results showed significant main effects of grade (*b* = -0.0781, *t* = -2.313, *p* = 0.024; *F* = 5.35, *p* = 0.024), i.e., higher grade was associated to faster first fixation duration, and of word frequency (*b* = 0.0142, *t* = 2.040, *p* = 0.045; *F* = 4.16, *p* = 0.045), i.e., higher word frequency was associated to longer first fixation. Base-word frequency and base-word category affected first fixation duration both as main effects (base frequency: *b* = -0.0274, *t* = -2.553, *p* = 0.013; *F* = 0.28, *p* = 0.60; base category: *b* = -0.2289, *t* = -2.667, *p* = 0.009; *F* = 7.11, *p* = 0.009) and in interaction with each other (*b* = 0.0460, *t* = 2.962, *p* = 0.004; *F* = 8.77, *p* = 0.004). In Model 2 proficiency was added to the list of covariates and the model fit significantly improved (AIC_model1_ = 6329.2, AIC_model2_ = 6322.0; *χ*^2^ = 9.213, *df* = 1, *p* = 0.002). In this model, the significance of grade effect disappeared, and proficiency emerged as the strongest predictor of first fixation (*b* = 0.0891, *t* = 3.074, *p* = 0.003; *F* = 9.45, *p* = 0.003). In the model run after removing grade factor, base-word category was involved in a four-way interaction with proficiency, word length, and word frequency (*b* = -0.033, *t* = -2.071, *p* = 0.038; *F* = 3.60, *p* = 0.058), and it also interacted with base-word frequency (*b* = 0.045, *t* = 2.535, *p* = 0.01; *F* = 5.84, *p* = 0.016). To better explore these interactions, conditional effects were decomposed (see Aiken and West, 1991). Models assessing effects of proficiency, word frequency, word length and base frequency were carried out on dataset split by base-word category.

For words derived from noun base (*N* = 2102), the three-way interaction within proficiency, word length, and word frequency was positive, and was far from a significance level (*b* = 0.0147, *t* = 1.286, *p* = 0.199; *F* = 1.65, *p* = 0.199). Neither two-way interactions nor main effect of word length reached a significance level. The final model (Table 3) showed that the lower proficiency was, the longer the first-fixation duration (*b* = 0.087, *t* = 3.28, *p* = 0.002; *F* = 11.38, *p* = 0.001), and word frequency had an inhibitory effect (*b* = 0.021, *t* = 2.09, *p* = 0.04; *F* = 4.62, *p* = 0.038). On the contrary, the main effect of base-word frequency was significant and higher frequency was associated with shorter first-fixation duration (*b* = -0.033, *t* = -2.78, *p* = 0.008; *F* = 7.35, *p* = 0.01). From a qualitative point of view, errors consisted of the production of a base word without suffix, e.g., ‘testa’ (head) instead of ‘test*ata*’ (headboard), ‘palazzo’ (palace, hall) instead of ‘palazz*etto*’ (sports center). So, we can hypothesize a strong competition between base- and whole-word representations in the early processing phases.

**Table 3:** Words derived from noun base: mixed-effects on *first-fixation duration.*

Random effects	Variance	*SD*			
Item (Intercept)	0.0004	0.0201			
Subjects (Intercept)	0.0335	0.1829			
Residual	0.3189	0.5646			

**Fixed effects**	**Estimate**	***SE***	***t*-Value**	***F-*Value**	***p-*Value**

(Intercept)	5.30147	0.05906	89.750		
Proficiency	0.08739	0.02665	3.282	11.388	0.001
Word frequency	0.02139	0.01027	2.088	4.62	0.038
Base-word frequency	-0.03259	0.01172	-2.783	7.35	0.01

For nouns derived from verb base (*N* = 1525), three-way interaction within proficiency, word length, and word frequency was negatively oriented and far from significance level (*b* = -0.0123, *t* = -1.102, *p* = 0.27; *F* = 1.22, *p* = 0.27). Also for these stimuli, neither two-way interactions nor word-length effects were significant, whereas a proficiency effect emerged (*b* = 0.1145, *t* = 4.03, *p* < 0.001; *F* = 15.37, *p* < 0.001) in the expected direction. However, differently from words derived from noun base, the word frequency effect was far from significance level (*b* = 0.006, *t* = 0.636, *p* = 0.53; *F* = 0.40, *p* = 0.52). The base-word frequency approached a significance level, but in the opposite direction from that observed in the other set of stimuli (*b* = 0.0217, *t* = 1.829, *p* = 0.067; *F* = 4.13, *p* = 0.04): higher base-word frequency was associated to slower first fixation. The null effect of word frequency suggests that, for these derived words, first fixation cannot be informative enough to activate any base+suffix representation, even though the base-word frequency effect suggests that verb-base recognition may occur, thus producing an inhibitory effect.

### Gaze Duration

Analyses on gaze duration were carried out on a total of 3904 observations, i.e., all words with at least 1 fixation, that were correctly pronounced: 2281 for noun-base targets and 1623 for verb-base targets.

Like for accuracy, the only significant difference in mean gaze duration was observed between 5^th^ and 3^rd^ grade children (Tukey’s HSD: *p* = 0.024). Fourth graders did not differ from 3^rd^ to 5^th^ graders (Table 4). Mean gaze duration for nouns derived from noun base was shorter than for nouns derived from verb base, but the difference did not reach a significance level (noun base: *M* = 953.1 ms, *SD* = 272.5; verb base: *M* = 1024.1 ms, *SD* = 261.6).

**Table 4:** Gaze duration (ms): descriptive statistics and comparison within grades.

*Grade*	*N*	*M*	*SD*	*Minimum*	*Maximum*	*F*	*p*
3^rd^	20	1158	314.2	728	1903	4.00	0.023
4^th^	22	1079	295.8	553	2415		
5^th^	20	838	472.0	506	1550		

Also for gaze duration, however, mixed-effects models, carried out to assess the main effects of grade, base-word category, word length, word frequency, root length and base frequency, and the interactions within these variables, offered a complex pattern of results. In Model 1, grade affected gaze duration in a three-way interaction with base category and whole-word frequency (*b* = -0.044, *t* = -2.31, *p* = 0.02; *F* = 5.62, *p* = 0.018). In Model 2, proficiency was added to the analysis, gaining an improvement in fit index (AIC_model1_ = 5932.0, AIC_model2_ = 5902.8; *χ*^2^ = 53.149, *df* = 1, *p* < 0.001). The main effect of proficiency was highly significant (*b* = 0.263, *t* = 4.124, *p <* 0.001; *F* = 34.2, *p* < 0.001), whereas all the effects involving grade became not-significant. Due to this lack of significance of grade factor, further analyses were carried out without the variable grade. In the final model, base-word category was involved in two three-way interactions: with proficiency and word frequency (*b* = 0.051, *t* = 3.31, *p* < 0.001; *F* = 10.98, *p* = 0.001), and with proficiency and base frequency (*b* = -0.037, *t* = -2.43, *p* = 0.015; *F* = 5.73, *p* = 0.017). Moreover, the three-way interaction within proficiency, word length, and word frequency was highly significant (*b* = -0.0268, *t* = -2.84, *p* = 0.005; *F* = 8.52, *p* = 0.003). Also in this case, in order to analyze lower-level interactions, further analyses were carried out separately for words derived from noun base and words derived from verb base, to decompose conditional effects.

For nouns derived from noun base (Table 5), mixed-effects model, tested with a model criticism procedure, showed a strong effect of proficiency (*b* = 0.2429, *t* = 4.30, *p* < 0.001; *F* = 18.39, *p* < 0.001), in the expected direction: when proficiency is lower, gaze duration is longer. The three-way interaction among proficiency, word length and word frequency reached significance level (*b* = -0.0263, *t* = -2.76, *p* = 0.006; *F* = 7.86, *p* < 0.01), as well as the embedded two-way interactions within proficiency and word length (*b* = 0.0936, *t* = 2.79, *p* = 0.005; *F* = 8.17, *p* < 0.01), and proficiency and word frequency (*b* = -0.0395, *t* = -3.85, *p* < 0.001; *F* = 12.9, *p* < 0.001).

**Table 5:** Words derived from noun base: mixed-effects on *gaze duration.*

Random effects	Variance	*SD*			
Item (Intercept)	0.0725	0.2693			
Subjects (Intercept)	0.0507	0.2254			
Residual	0.2384	0.4883			

**Fixed effects**	**Estimate**	***SE***	***t*-Value**	***F-*Value**	***p-*Value**

(Intercept)	6.9050	0.1977	34.930		
Proficiency	0.2429	0.0564	4.297	18.39	<0.001
Word length	0.0213	0.0102	2.088	1.89	0.18
Word frequency	-0.0747	0.0416	-1.793	3.23	0.08
Base frequency	0.0056	0.0392	0.143	0.03	0.86
Proficiency^∗^Base frequency	0.0243	0.0097	2.504	5.27	0.02
Word length^∗^Word frequency	-0.0334	0.0387	-0.862	0.68	0.41
Proficiency^∗^Word length	0.0936	0.0332	2.787	8.17	<0.01
Proficiency^∗^Word frequency	-0.0395	0.0102	-3.854	12.9	<0.001
Proficiency^∗^Word length^∗^Word frequency	-0.0263	0.0094	-2.755	7.86	<0.01

**FIGURE 1:**
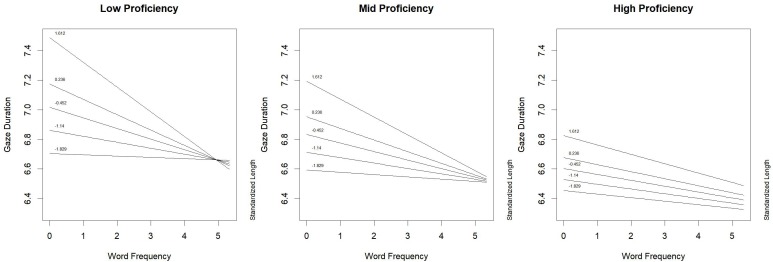
Words derived from noun base: effect of proficiency *by* word frequency *by* word length interaction on gaze duration.

Following Bauer and Curran (2005) suggestions, interactions were explored by graphical techniques. Figure 1 shows the three-way interaction: when reading proficiency was low (left panel of Figure 1), the inhibitory effect of word length (from -1.89 to 1.612 *z*, corresponding to quantiles of the standardized length in letters) on gaze duration was mitigated by word frequency. In fact, such inhibitory effect was very evident for low frequency words, and tended to disappear for high-frequency words. This result suggests that the lower the reading proficiency the higher the probability that low-frequency words are read through fine-grained orthography representations (Grainger and Ziegler, 2011), thus being negatively affected by length in letters. On the contrary, high-frequency words are likely to activate coarse-grained representations, which are less affected by word length than the fine-grained code. Such modulation becomes less evident with increasing proficiency. When proficiency was high (right panel of Figure 1), the two opposite effects of word length and word frequency were independent: a frequency effect was observed, but the inhibitory effect of word length was quite similar for all words, irrespective of their lexical frequency. In this case, it can be assumed that both low- and high-frequency words are processed mainly through coarse-grained orthographic representations.

**FIGURE 2:**
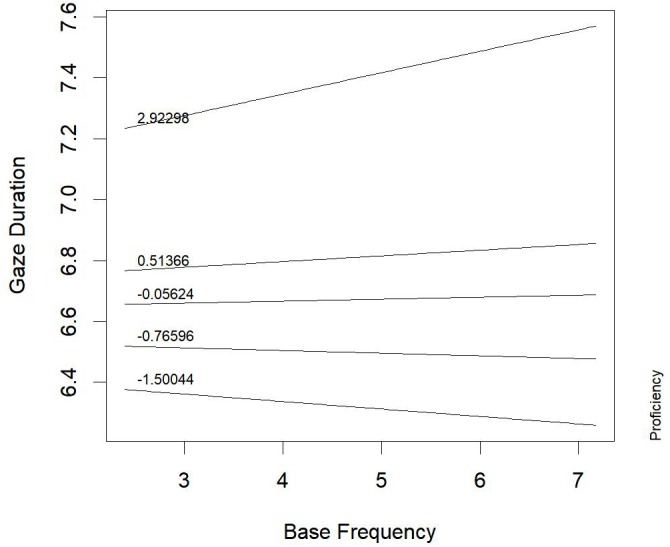
Words derived from noun base: proficiency *by* base-frequency interaction effect on gaze duration.

Figure 2 represents the interaction between proficiency and base frequency (*b* = 0.0243, *t* = 2.50, *p* = 0.02; *F* = 5.27, *p* = 0.02): in low-proficiency children (with scores of about 2.92 *z*, corresponding to quantiles of the factor scores on reading time measure), the higher the base frequency, the longer the gaze duration. This result suggests that base-word activation can lead to time-consuming processing of the derived words in less proficient readers, maybe for the conflict between base- and whole-word representations, which was noticed in the early stages of processing, through the analysis of first-fixation duration. This conflict seems to still affect reading at later processing stages, but only for less proficient readers. Skilled readers did not show such an effect anymore, suggesting that the initial conflict is quickly solved by these children.

**FIGURE 3:**
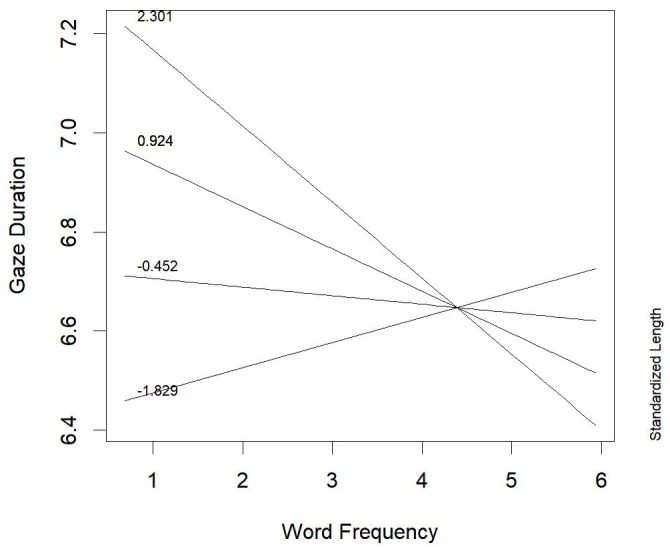
Words derived from verb base: word frequency *by* word length interaction effect on gaze duration.

Also for words derived from verb base (Table 6), the main effect of proficiency on gaze duration was very strong (*b* = 0.271, *t* = 3.52, *p* < 0.001; *F* = 12.38, *p* < 0.001): the lower the proficiency, the longer the gaze duration. The three-way interaction within proficiency, word length, and word frequency did not reach a significance level (*b* = -0.0135, *t* = -1.40, *p* = 0.16; *F* = 1.95, *p* = 0.16), but was in the same direction of the effect observed in nouns derived from noun bases. The two-way interactions between proficiency and word length (*b* = 0.031, *t* = 0.92, *p* = 0.34; *F* = 0.84, *p* = 0.34), and between proficiency and word frequency (*b* = 0.013, *t* = 1.11, *p* = 0.27; *F* = 1.23, *p* = 0.27) were not significant. Only the two-way interaction between word length and word frequency emerged, irrespective of children’s proficiency (*b* = -0.053, *t* = -2.27, *p* = 0.03; *F* = 5.16, *p* = 0.03; Figure 3): for low- to middle-frequency words (until up to about 100 occurrences/million), the inhibitory effect of word length on gaze duration was stronger than the facilitative effect of word frequency.

**Table 6:** Words derived from verb base: mixed-effects on *gaze duration.*

Random effects	Variance	*SD*			
Item (Intercept)	0.0215	0.1468			
Subjects (Intercept)	0.0461	0.2148			
Residual	0.2446	0.4946			

**Fixed effects**	**Estimate**	***SE***	***t*-Value**	***F-*Value**	***p-*Value**

(Intercept)	6.912	0.1738	39.766		
Proficiency	0.2714	0.0771	3.519	12.38	<0.001
Word length	0.2234	0.0803	2.781	7.73	0.01
Word frequency	-0.0383	0.0286	-1.341	1.80	0.19
Base frequency	0.0084	0.0278	-0.304	0.09	0.76
Proficiency^∗^Base frequency	-0.0134	0.0117	-1.139	1.30	0.25
Word length^∗^Word frequency	-0.0527	0.0232	-2.271	5.16	0.03
Proficiency^∗^Word length	0.0306	0.0335	0.916	0.84	0.35
Proficiency^∗^Word frequency	0.0130	0.0117	1.109	1.23	0.27
Proficiency^∗^Word length^∗^Word frequency	-0.0135	0.0097	-1.398	1.95	0.16

This result suggests that for words derived from verb base the fine-grained code is the most used, irrespective of reading proficiency. Only for high-frequency words, the role of word length got smaller and changed its direction. The latter result might be explained by the presence, in the ending of high-frequency long words derived from verb base, of the longest and most productive suffix used, in Italian, in deriving nouns from verbs, i.e., *-zione* (e.g., *operazione*, operation; *produzione*, production; *costruzione*, construction, etc.). In this case the base+suffix combination is quite predictable. Thus, gaze duration was shorter for such long words than for short high-frequency derived words ending with less productive and short suffixes like -*nza* (e.g., *partenza*, departure; *speranza*, hope).

For this set of stimuli, base-word frequency did not affect gaze duration either as main effect (*b* = -0.008, *t* = -0.304, *p* = 0.76; *F* = 0.09, *p* = 0.76) or in interaction with proficiency (*b* = -0.013, *t* = -1.14, *p* = 0.25; *F* = 1.30, *p* = 0.25).

## Discussion

In a previous study on 4^th^ and 5^th^ grade children, participants were asked to read aloud nouns derived from noun base and nouns derived from verb base (Traficante et al., 2014): morpho-lexical effects on RTs emerged for nouns derived from verb base, whereas only length in letters affected latencies for nouns derived from noun base. However, for both types of stimuli, base- and whole-word frequency affected accuracy: the higher the frequency measures, the more accurate the children’s performance. Data on latency was interpreted as evidence for the peculiarity of verb bases, which are more likely to be used as a headstart for naming than noun bases, due to the distributional properties of verb paradigms. That evidence came from latency measure and informed us on the time needed by the reader for finding, in the string of letters, some familiar letter chunk to start the stimulus pronunciation. Morpho-lexical effects on accuracy observed in reading not only nouns derived from verb base, but also nouns derived from noun base, suggested that even noun base might be involved in the recognition process. In the present work we presented the same target words selected in the previous study, embedded in sentences, and used eye-movement recording to get clues on the whole processing of derived words, and not only on the pronunciation onset. Data from literature showed that eye-movement recording allows one to reveal the reader’s behavior from early processing (first-fixation duration) to the final word recognition (gaze duration). Studies in the field showed that eye-movements are guided by “the big three” (i.e., word frequency, word length, and predictability in the context), and by the morphemic parsing procedure. Moreover, Häikiö et al.’s (2011) study of eye-movements in Finnish children showed that the use of morphemic constituents in that population can be affected by the reading proficiency.

### First-Fixation Duration

Data from first-fixation duration showed a different pattern of results for words derived from noun bases and words derived from verb bases, but offers a picture of early processing which is quite different from evidence obtained with latency measure in the previous work. For nouns derived from noun bases, both base-word frequency and whole-word frequency affected children’s first-fixation duration, but in the opposite direction. Base-word frequency exerted a facilitative effect, as the higher the frequency, the faster the first-fixation duration: so children seemed to gain advantage from the base activation. However, the higher the whole-word frequency, the longer the first-fixation duration. It seems that children, when the target is a high-frequency word, are faced with two conflicting representations: the base word and the whole word, which, in the case of noun-base targets, are both equally consistent with the grammatical and semantic constraints related to the sentence context. This condition of uncertainty seems to lead to longer first fixation. Erroneous productions by children supported this interpretation, as almost all errors consisted in productions of the base word with the omission of the suffix (e.g., ‘mattina,’ instead of ‘mattin*ata*,’ morning; ‘camino,’ chimney, instead of ‘camin*etto*,’ fireplace, etc.).

This result, along with data found by Amenta et al. (2015), suggested that first-fixation duration might be sensitive to possible competition, induced by sentence context, between base-word and whole-word representations. The complex interplay between base- and whole-word frequency observed in first-fixation duration might explain the null effect of morpho-lexical features on latency measure, obtained in the previous study, as the result of a competition between two alternative lexical representations, which leads to a reduction of frequency effects on RTs. However, these results from eye-movement recordings showed that both (base- and whole-word) representations come into play, as accuracy data from the previous study has suggested.

As for words derived from verb base, first fixation was influenced by reader’s proficiency, as the lower proficiency, the slower the first fixation, and by base frequency, which exerted an inhibitory effect. Null-effect of whole-word frequency can be interpreted as the readers’ difficulty in accessing the base+suffix combination in the very early processing phases, and can be explained in light of the experimental conditions. In fact, it can be assumed that, during first fixation, parafoveal information (see Reichle et al., 2013 for perceptual span in children) concerning word length gives the reader the cue that the target must be a long word, embedding the base word which is situated at the beginning of the letter string. In the case of a noun base, as discussed above, a competition between base word and whole word is likely to occur. In the case of a verb base, the syntactic context seems to determine a condition that is similar to GCST. In fact, all targets were nouns and the sentences were made up with a plain syntactic structure, according to usual word order in the Italian language, which requires a noun in the target position. When the reader detects a verb base at the beginning of the target word, a conflict is likely to occur between expected noun base and actual verb base. The inhibitory effect from this conflict seems stronger for high-frequency bases. In the derivation task used in Silveri and colleagues studies (Di Tella et al., 2018; Silveri et al., 2018), healthy adults and patients with Parkinson’s Disease were asked to read a verb base (e.g., ‘osservare,’ to observe) on a computer screen and to retrieve the noun derived from that base (e.g., ‘osservazione,’ observation). It was a very difficult task even for adults in a healthy-control group. Those results were consistent with data from the fMRI study by Berlingeri et al. (2008) on healthy undergraduate students, that showed slower RTs for nouns than for verbs in GCST. In all those studies, results were interpreted as evidence that deriving a noun from a verb involves complex selection and inhibition processes, due to the large number of derived and inflected words sharing the same verb base. So, the lack of a significant word frequency effect on first-fixation duration that emerged from the present study can be conceivable for primary school children, who during early phases of processing might not have automatic access to the lexical representations involved in such a difficult computation.

### Gaze Duration

Differences between words derived from noun base and words derived from verb base could also be observed in gaze duration. For the first category of stimuli (i.e., derived words with noun base), readers’ proficiency played an important role in determining the impact that word length and word frequency exerted on children’s eye movements. For low-proficiency children, the inhibitory effect of word length was stronger for low- rather than for high- frequency words. This result suggested that the processing of low-frequency words is carried out through time-consuming fine-grained orthographic representations (Grainger and Ziegler, 2011). On the contrary, for high-frequency words, lexical representations were likely to be accessed through coarse-grained orthographic representations, thus reducing the impact of word length. It is worth noting, however, that the effect of word length on gaze duration was also modulated by proficiency: it was smaller for high-proficiency children than for low-proficiency children. Moreover, for high-proficiency children length effect was independent from word frequency effect. This result suggests that low-frequency words are likely to be recognized by skilled children through the same procedure applied for high-frequency words, i.e., coarse-grained representations. This pattern of results mirrors a different degree of automatization in accessing lexical representations and a different use of sublexical/morpholexical procedure in reading aloud, according to different degrees of reading proficiency (Grainger and Ziegler, 2011). Difficulty of low-proficiency children in word recognition turned out also in the inhibitory effect of the base word, that can be observed as the proficiency decreases. This effect is consistent with Beyersmann et al. (2015) results, as in masked-priming paradigm low-proficiency children (grades 2–5) showed inhibition in finding a real stem in a non-word prime stimulus, whereas high-proficiency children showed facilitation.

Evidence from first fixation duration along with data from gaze duration suggested that, in early processing, when derived nouns are presented in a sentence context, noun-base representation (which is much more frequent than whole-word representation: see Table 1) can be a useful headstart for all children. However, its positive effect could be reduced by the competition with whole-word representation. Uncertainty between the two representations seems to be resolved in later stages of processing by skilled readers, whose gaze duration is affected only by whole-word frequency (the higher the word frequency, the faster gaze duration is) and length (the longer the word, the longer the gaze duration). Word length had a strong effect also on gaze duration in children with low proficiency in reading, mainly for low-frequency words. Moreover, for these children, the competition of base representation could be observed still in a late processing phase, as the higher the base frequency, the longer the gaze duration.

As for words derived from verb bases, all children, irrespective of their proficiency in reading, showed an interplay between length and word frequency effects: length effect on gaze duration was much stronger for low-frequency words, suggesting a massive use of fine-grained orthography for these words. However, it is worth noting that the higher the word frequency (i.e., frequency of base+suffix combination), the smaller the length effect was, until an inversion of the effect for highest frequency words was observed. The longest and most frequent words were associated with the shortest gaze duration: this effect can be due to the presence, in these words, of a long and very productive suffix (e.g., -*zione*, -tion; see Supplementary Material), which can work as a very useful chunk.

Summing up the results from the two procedures we used to contrast noun and verb bases, i.e., reading words in isolation (Traficante et al., 2014) and reading words in a sentence context (the present study), it seems that the effect of probabilistic features may be modulated by the processing context. As for verb bases, the effects of both base and word frequency on latency and accuracy, when the stimulus is presented in isolation (Traficante et al., 2014), suggest that the onset of pronunciation starts as soon as the base is recognized, and the pronunciation of the whole word can be computed as the base+suffix combination. The inhibitory effect of base frequency on first-fixation duration, observed in the present study, leads to hypothesize that the information acquired with the first fixation does not promote the onset of pronunciation, when the stimulus is embedded in a sentence calling for a noun in the target-word position: on the contrary, the inconsistency between the early detected verb base and the expected noun base is likely to engage the reader in difficult selection and inhibition processes, similar to those investigated in other studies in Italian adults (Marangolo et al., 2006; Berlingeri et al., 2008; Di Tella et al., 2018; Silveri et al., 2018). Only when the whole base+suffix combination has been accessed in later processing (as tapped by gaze duration), the reader can gain advantage from word frequency, with a reduction of inhibitory length effect. The inversion of the length effect for the most frequent words suggests that the long suffix -*zione* is a more useful chunk than the shorter suffix -*nza*, and support the hypothesis of the use of fine-grained codes in recognition of words derived from verb base.

As for words derived from noun bases in isolation (Traficante et al., 2014), the lack of lexical effects on latency in reading aloud was an unexpected result and we interpreted it by assuming that morphemic constituents, in those stimuli, are not informative enough to influence RTs in word processing. However, both base and whole-word frequency affected accuracy in reading these words: so, there was evidence for activation of morpho-lexical representations in processing words derived from noun base. Data from the present study offers a different pattern of results: when the sentence context leads to foreseeing a noun, first-fixation duration shows that noun base is likely to be already activated since early processing steps, but the gain from this activation is reduced by the competition of whole-word representation, as eye-movement analyses proved. The base is shorter and more frequent than the derived word, and shares the core-meaning with it, so the base word may be a good option, that fits with the features of the expected target, and may play the role of competitor of the derived word, in particular when reading proficiency is low. Data from gaze duration shows that in late processing only word length and word frequency affects the eye-movement of skilled readers, whereas base representation seems to be still a competitor of whole-word representation only in low proficiency children.

To conclude, the pattern of results shows a complex picture, in which the relation between base- and whole-word representations may be not only complementary, as in the case of reading aloud single words, but also competitive, as in the case of reading aloud derived words in a sentence context. Moreover, our data, along with results from Finnish and French studies, suggests that processing morphologically complex words is not influenced by grade, i.e., by explicit learning, but by reading skills that have been referred to several individual’s features, such as visual perceptual span (Bosse and Valdois, 2009), decoding and lexical access speed (Wolf and Bowers, 1999; Blythe and Joseph, 2011; Zoccolotti et al., 2014), and sensitivity to semantic and distributional properties of language (Marinelli et al., 2017) (implicit learning processes).

Finally, the comparison between results from different experimental procedures, i.e., reading words in isolation (Traficante et al., 2014) vs. reading words in a sentence context (this research), involving the same stimuli, offered an interesting insight on the role of syntactic context in word recognition and, in particular, in recognition of morphemic constituents. Development of new measures on distributional properties of words in different syntactic and semantic contexts might offer useful means to collect new evidence on the development of probabilistic strategies in reading acquisition, and for modeling morphemic parsing in young readers with different proficiency levels.

## Ethics Statement

This study was carried out in accordance with the recommendations of the Psychological Research Institutional Review Board of Catholic University of Milan, with written informed consent from all subjects’ parents. All subjects’ parents gave written informed consent in accordance with the Declaration of Helsinki. The protocol was approved by the Psychological Research Institutional Review Board – Catholic University of Milan.

## Author Contributions

All authors listed have made a substantial, direct and intellectual contribution to the work, and approved it for publication.

## Conflict of Interest Statement

The authors declare that the research was conducted in the absence of any commercial or financial relationships that could be construed as a potential conflict of interest.

## Footnotes

1 In GCST, participants are presented either with a verb (e.g., to observe) and are asked to produce the corresponding derived noun (e.g., observation), or with a noun (e.g., production) and are required to produce the verb base (e.g., to produce).

2 Correlation coefficient between grade and proficiency is 0.448, so it was possible to include both variables in the same model.

3 Akaike Information Criterion (AIC): AIC = 2k – 2ln(L), where k = number of parameters and L = maximum likelihood.

4 Remember that proficiency measure is negatively oriented.

## Supplementary Material

TABLE S1 Target words and stimuli list.
